# Acute exacerbations of chronic obstructive pulmonary disease are associated with decreased CD4+ & CD8+ T cells and increased growth & differentiation factor-15 (GDF-15) in peripheral blood

**DOI:** 10.1186/s12931-015-0251-1

**Published:** 2015-08-05

**Authors:** Christine M. Freeman, Carlos H. Martinez, Jill C. Todt, Fernando J. Martinez, MeiLan K. Han, Deborah L. Thompson, Lisa McCloskey, Jeffrey L. Curtis

**Affiliations:** Research Service and Pulmonary & Critical Care Medicine Section, Medicine Service, VA Ann Arbor Healthcare System, Ann Arbor, MI 48105 USA; Pulmonary & Critical Care Medicine Section, Medicine Service, VA Ann Arbor Healthcare System, Ann Arbor, MI 48105 USA; Graduate Program in Immunology, University of Michigan, Ann Arbor, MI 48109 USA; Pulmonary & Critical Care Medicine Division, Department of Internal Medicine, University of Michigan Health System, Ann Arbor, MI 48109 USA; Department of Veterans Affairs Healthsystem, Pulmonary and Critical Care Medicine Section (506/111G), 2215 Fuller Road, Ann Arbor, MI 48105-2303 USA

**Keywords:** Human, Flow cytometry, Blood biomarkers, Sputum, Exacerbation, COPD

## Abstract

**Background:**

Although T cells, especially CD8+, have been implicated in chronic obstructive pulmonary disease (COPD) pathogenesis, their role during acute exacerbations (AE-COPD) is uncertain.

**Methods:**

We recruited subjects with COPD and a history of previous AE-COPD and studied them quarterly to collect blood and spontaneously expectorated sputum while stable. During exacerbations (defined by a change in symptoms plus physician diagnosis and altered medications), we collected blood and sputum before administering antibiotics or steroids. We used flow cytometry to identify leukocytes in peripheral blood, plus Luminex® analysis or ELISA to determine levels of inflammatory biomarkers in serum and sputum supernatants.

**Results:**

Of 33 enrolled subjects, 13 participated in multiple stable visits and had ≥1 AE-COPD visit, yielding 18 events with paired data. Flow cytometric analyses of peripheral blood demonstrated decreased CD4+ and CD8+ T cells during AE-COPD (both absolute and as a percentage of all leukocytes) and significantly increased granulocytes, all of which correlated significantly with serum C-reactive protein (CRP) concentrations. No change was observed in other leukocyte populations during AE-COPD, although the percentage of BDCA-1+ dendritic cells expressing the activation markers CD40 and CD86 increased. During AE-COPD, sICAM-1, sVCAM-1, IL-10, IL-15 and GDF-15 increased in serum, while in sputum supernatants, CRP and TIMP-2 increased and TIMP-1 decreased.

**Conclusions:**

The decrease in CD4+ and CD8+ T cells (but not other lymphocyte subsets) in peripheral blood during AE-COPD may indicate T cell extravasation into inflammatory sites or organized lymphoid tissues. GDF-15, a sensitive marker of cardiopulmonary stress that in other settings independently predicts reduced long-term survival, is acutely increased in AE-COPD. These results extend the concept that AE-COPD are systemic inflammatory events to which adaptive immune mechanisms contribute.

**Trial registration:**

NCT00281216, ClinicalTrials.gov.

**Electronic supplementary material:**

The online version of this article (doi:10.1186/s12931-015-0251-1) contains supplementary material, which is available to authorized users.

## Introduction

Chronic obstructive pulmonary disease (COPD) is a progressive, debilitating and highly prevalent condition that is believed to result from an abnormal response to inhaled oxidants and which is characterized by lung destruction, airways remodeling, and mucus hypersecretion. COPD is punctuated by episodic worsening in respiratory function and symptoms, termed acute exacerbations of COPD (AE-COPD). These events are associated with accelerated lung function decline, reduced quality of life [1, 2], plus increased mortality, which is appreciable for events requiring hospitalization and which persists for at least five years [3, 4]. AE-COPD also drive the majority of the economic burden of this common disease [5]. Preventing these expensive and potentially lethal events should be a major public health goal.

Despite this great impact of AE-COPD, understanding of the responsible pathogenic mechanisms remains incomplete. There is consensus that a plurality of AE-COPD are associated with isolation of respiratory microbes, with some contribution from environmental triggers [6–8], and some events lacking evidence of inflammation, currently of incompletely understood etiology [9]. However, the acute beneficial role of systemic steroid therapy suggests that overly-exuberant inflammation, rather than simply presence of microbes, might determine symptomatology in most AE-COPD. Hence, continued investigation of inflammatory mechanisms during AE-COPD remains important.

We have hypothesized that AE-COPD would be associated with systemic evidence of inappropriate immune activation, especially of the adaptive immune system [10]. Lung T cells, especially CD8+ T cells, have been implicated in COPD pathogenesis [11–13], but data on their role in AE-COPD are limited. During AE-COPD, numbers of lymphocytes increase in both sputum and airway biopsies [14–16] and a CD8+ type-2-mediated immune reaction has been identified in sputum [17]. However, in the peripheral blood, a practical sample for monitoring systemic responses, there are few data during AE-COPD on the activation status and phenotype of immune cells or on their correlation with circulating biomarkers. The lack of universally-accepted animal models of AE-COPD necessitates use of human samples to address this question.

To that end, we designed a prospective cohort study to investigate subjects during the stable state and at onset of AE-COPD. Peripheral blood was used for both immunophenotyping of various leukocytes populations and to analyze 41 analytes in serum. Sputum supernatants were used to analyze 36 analytes. Our results provide novel data supporting AE-COPD as systemic inflammatory events to which T cells contribute, and which even when not requiring hospitalization, are associated with pulmonary vascular stress.

## Materials & methods

### Study design

This observational longitudinal cohort study aimed to collect biomarkers of AE-COPD, for paired, within-subject comparison with samples collected at enrollment during clinical stability. Accordingly, we recruited participants at high risk of repeated AE-COPD who agreed to return to the study sites during suspected exacerbations. Individual subjects were followed for up to 3 years.

### Ethics, consent and permissions

Subjects were recruited both from Pulmonary and General Medicine clinics at VA Ann Arbor Healthcare System (VAAAHS) and the University of Michigan Health System (UMHS) and by use of additional media (flyers, UMHS website for clinical research volunteers). Studies and consent procedures were performed in accordance with the Declaration of Helsinki and were approved by each site’s Institutional Review Board. Written consent to participate was obtained before any study procedures. All participants understood the purpose of the study and consented to publication of individual data. The study period was from December 2006 until March 2010.

### Subject population & participation

Subjects were enrolled while clinically stable ≥6 weeks post AE-COPD or other use of antibiotics. Inclusion criteria were a diagnosis of COPD by GOLD guidelines [18]; a post-bronchodilator forced expiratory volume in one second (FEV_1_) of ≤ 70 % predicted; cigarette smoking exposure ≥ 20 pack years; 40 years ≤ age ≤ 80 years; daily productive cough ≥3 months annually for two consecutive years; and at least one AE-COPD requiring medical attention and a medication change (addition of oral steroids, antibiotics or both) annually for the previous three years.

Exclusion criteria included unstable heart disease; illnesses anticipated to result in death within two years; prednisone > 20 mg per day; asthma, cystic fibrosis, clinically significant bronchiectasis, lung cancer, or other inflammatory or fibrotic lung diseases.

Participants underwent spirometry, prospectively-collected questionnaires, clinical evaluation by a pulmonologist, posterior-anterior and lateral chest radiographs and collection of peripheral blood and spontaneously expectorated sputum at enrollment and during unscheduled visits when they sought medical attention for presumptive AE-COPD. During scheduled follow-up visits when participants were clinically stable, study coordinators performed identical data collection and evaluation (omitting radiographs).

AE-COPD was defined as a change above baseline in respiratory symptoms (increased dyspnea, cough, and sputum volume or purulence) plus addition by a study physician of antibiotics, oral steroids or both, based on review of history, physical examination and chest radiographs to exclude pneumonia. A diagnosis of AE-COPD was made prior to collection of samples (so study physicians had no knowledge of biomarker results, including serum CRP) and of the Breathlessness, Cough, and Sputum Scale questionnaire. Only if the unscheduled visit was determined clinically to be an AE-COPD meriting treatment using oral steroids, antibiotics or both, were peripheral blood and spontaneously expectorated sputum specimens collected, which occurred before initiating any new medications.

### Sample collection & processing

Peripheral blood was collected into two Vacutainer blood collection tubes (Becton Dickinson, Franklin Lakes, NJ). One containing heparin was used for whole blood flow cytometric analyses; the other without additives was allowed to clot for 30 min at room temperature before centrifugation and then serum was collected into cryovials and stored at −80 °C until analysis.

Spontaneously expectorated sputum was immediately transferred to the laboratory, on ice, for processing. Dense, non-liquid portions of sputum were selected and weighed. Sputolysin® (EMD Millipore, Billerica, MA) was prepared by dissolving a 1 mL vial in 9 mL distilled water. The prepared Sputolysin was added at 2x the weight of the sputum and incubated in a 37 °C waterbath with vortexing every 5 min until the sample became viscous. An equal volume of PBS was added and the mixture was filtered through a 48 μm nylon gauze mesh filter, centrifuged and the supernatant was stored at −80 °C until analysis.

### Flow cytometry

Whole blood was dispersed into tubes (100 μL each) containing antibodies and incubated for 25 min, protected from light, with shaking. BD FACS™ Lysing Solution (Becton Dickinson) was used to lyse erythrocytes according to manufacturer’s instructions. We used monoclonal antibodies from a variety of vendors as indicated in Additional file 1: Supplemental Materials & Methods. We assessed viability in all experiments using a Live/Dead Fixable Near-IR Dead Cell Stain Kit for 633 nm excitation (Invitrogen, Carlsbad, CA). Immediately after staining, cells were fixed by addition of staining buffer containing 2 % paraformaldehyde; tubes were then stored at 4 °C in a rack wrapped in aluminum foil until analyzed.

Staining was analyzed on a LSR II flow cytometer (BD Bioscience, San Jose, CA), equipped with four lasers as described in detail [19]. Data were collected using FACSDiva software (BD Biosciences) with automatic compensation and were analyzed using FlowJo software (Tree Star, Ashland, OR). We collected at least 10,000 viable CD45+ events per sample in each experiment. Details of our gating strategy are provided in Additional file 1: Supplemental Materials & Methods, and have recently been published together with representative raw data [20].

### Serum & sputum protein measurements

Serum and sputum samples were stored at −80 °C until all could be analyzed simultaneously. We used two methodologies to analyze protein concentrations. The majority of analytes were measured using a Luminex 200 system® (Luminex Corporation, Austin, TX), according to the manufacturer’s instructions. We used multiplex bead sets from a variety of vendors as indicated in Additional file 1: Supplemental Materials & Methods. For four analytes (GDF-15, IL-18, IL-23p19 and IFN-β), multiplex beads were not available; instead, ELISAs were used according to manufacturers’ instructions.

### Statistical analyses

We used GraphPad Prism 6.0 (GraphPad Software, Inc., La Jolla, CA) on a Macintosh Quad-Core Intel Xeon computer running OS 10.8.3 (Apple; Cupertino, CA). We used Wilcoxon matched-pairs signed rank test to look for differences between stable and exacerbation visits. A two-tailed *p* value of < 0.05 was considered to indicate significance. Spearman nonparametric correlation coefficient was used.

## Results

### Patient enrollment and completion

We enrolled 33 COPD subjects; six completed only the baseline visit and were excluded. Another 14 subjects completed multiple visits in the stable state; however, 13 did not complete an AE-COPD visit, despite an interval self-reported AE-COPD, and were excluded. A single subject did not have any AE-COPD. The remaining 13 subjects participated in multiple stable visits and had ≥1 AE-COPD visit, yielding 18 total events with paired stable and AE-COPD data. Age, sex, smoking history, spirometry, number of AE-COPD, and inhaled corticosteroid (ICS) use for each of the 13 subjects are shown (Table 1). All events were treated successfully in the outpatient setting.

**Table 1:** Summary of demographics, spirometry, smoking history and medications^1^

Age (years)	Sex	FEV1 (% predicted)	Smoking (pack-years)	Smoking status	# of AE-COPD during enrollment	ICS use (Y/N)	Home oxygen (Y/N)
67	M	52	108	Active	1	Y	N
74	F	51	50	Former^2^	3	Y	N
73	M	50	115	Former	1	Y	Y
59	M	47	18	Former	1	Y	Y
72	M	36	39	Former	2	Y	Y
66	F	33	104	Active	1	Y	Y
72	M	31	120	Active	1	Y	Y
66	M	29	40	Active	1	N	N
77	M	28	50	Former	1	Y	N
58	M	26	25	Former	1	Y	N
65	M	21	40	Former	1	Y	Y
67	M	20	84	Active	1	Y	Y
69	M	14	98	Former	3	Y	Y
68.1 ± 5.6^1^	11/2	33.7 ± 12.7	68.5 ± 36.9	8/5	1.38 ± 0.77	12/1	8/5

^1^Data are presented as average ± SD, except for sex ratios (M/F), smoking status (Former/ Active) and ICS use (Yes/No); M, male; F, female; ICS, inhaled corticosteroids; ^2^former smokers were defined as having quit for more than six months

Among the 18 captured AE-COPD events, 14 occurred 2–13 months after the preceding stable visit (5.3 ± 3.2 months, mean ± SD). However, in the other four, the AE-COPD occurred within one week after a scheduled stable visit. Given the possibility that data were not collected during a period of true stability, in these analyses we instead used paired data from a subsequent scheduled stable visit 2–5 months after the AE-COPD.

### Exacerbations resulted in increased symptom scores and elevated CRP levels

**Fig. 1:**
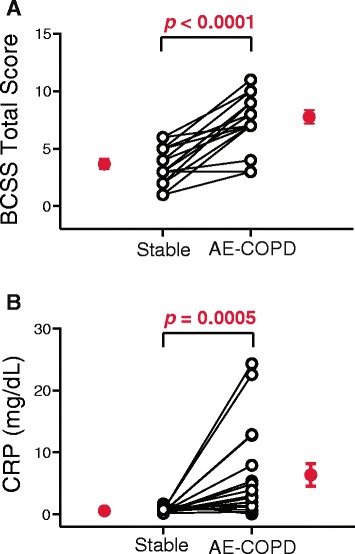
AE-COPD confirmed by increases in BCSS total score and serum CRP levels. AE-COPD events were identified by physician diagnosis (after exclusion of pneumonia by chest radiograph) plus the clinical decision to prescribe oral steroids or antibiotics. Although these decisions were blinded to biomarker results, the diagnosis of AE-COPD was retrospectively confirmed by highly significant increases in (**a**) BCSS total scores and (**b**) serum CRP levels. Open circles represent individual subjects; mean ± SEM of the grouped data are shown by the single red circles. The Wilcoxon matched-pairs signed rank test was used to determine significant differences between visits

All decisions to treat and hence to collect data at an AE-COPD visit were made by clinicians blinded to data from study questionnaires or biomarkers. However, as an independent confirmation that subjects were experiencing an actual AE-COPD, we retrospectively evaluated the severity of respiratory symptoms using the Breathlessness, Cough, and Sputum Scale (BCSS) [21]. The BCSS is based on a three-item questionnaire rating breathlessness, cough and sputum on a scale of 0 (no symptoms) to 4 (severe symptoms), which are summed. BCSS total score significantly increased during AE-COPD (Fig. 1a), from a median of 3.5 during stable visits to 8.0 during AE-COPD.

A study published shortly before the launch of ours, which assessed the ability of 36 different biomarkers to confirm the presence of AE-COPD and to predict their severity, found CRP to be the most selective analyte [22]. Serum CRP levels in our subjects, which were measured by the clinical laboratories, were also significantly increased during AE-COPD (Fig. 1b) (stable, 0.58 ± 0.40 mg/dL vs. AE-COPD, 6.4 ± 7.5 mg/dL; mean ± SD). In our study, BCSS correlated with CRP levels (*r* = 0.43, *p* = 0.011). Collectively, these findings provide additional evidence that these events were valid AE-COPD.

### Peripheral blood CD4+ and CD8+ T cells decreased during AE-COPD

**Fig. 2:**
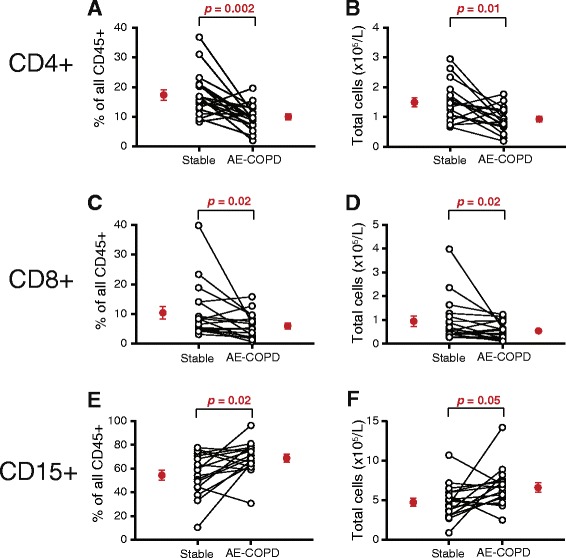
Peripheral blood CD4+ and CD8+ T cells decreased during AE-COPD while CD15+ granulocytes increased. Peripheral blood was collected during stable visits and during AE-COPD, prior to medication (*n* = 18 paired samples). Cells were stained and analyzed by flow cytometry. Results are shown for the individual cell type as a percentage of all CD45+ leukocytes (**a**, **c**, **e**) and as absolute cell numbers per μL (**b**, **d**, **f**). A, B. CD4+ T cells. C, D. CD8+ T cells. E, F. CD15+ cells; note difference in scale of panels E & F. Open circles represent individual subjects; mean ± SEM of the grouped data are shown by the single red circles. The Wilcoxon matched-pairs signed rank test was used to determine significant differences between visits

CD4+ T cells and CD8+ T cells significantly decreased in the blood during AE-COPD, relative to the paired stable visit (Fig. 2a-d). This change occurred both as a percent of all CD45+ cells (CD4+ T cells: stable, 17.4 ± 1.7 % of CD45+ leukocytes vs. during AE-COPD, 10.0 ± 4.2 %; mean ± SEM; *p* = 0.002) (CD8+ T cells: stable, 10.4 ± 2.2 % vs. AE-COPD, 5.9 ± 1.0 %; *p* = 0.02) and in absolute numbers. By Spearman correlation analyses, CD4+ and CD8+ T cells correlated significantly and inversely with serum CRP, both relatively (percentage CD4+ T cells: *r* = −0.74, *p* < 0.0001; percentage CD8+ T cells: *r* = −0.46, *p* = 0.005) and as absolute cell numbers (CD4+ T cells: *r* = −0.60, *p* = 0.0001; CD8+ T cells: *r* = −0.41, *p* = 0.013).

We did not see changes during AE-COPD in the distribution in peripheral blood of B cells, NK cells or any of the three types of DCs, in either relative or absolute terms. However, CD15+ granulocytes increased significantly (Fig. 2e-f), both as a percent of CD45+ cells (stable, 54.5 ± 4.3 % of CD45+ cells vs. AE-COPD, 68.9 ± 3.3 %) and in absolute numbers. CD15+ cells correlated directly with serum CRP (percentage CD15+ cells, *r* = 0.59, *p* = 0.0003; absolute CD15+ cells, *r* = 0.49, *p* = 0.003).

Because the flow cytometric staining used in this study had not been designed to distinguish neutrophils from eosinophils, we retrospectively examined data from the differential cell counts performed by the clinical laboratories. There were no significant changes in eosinophils during AE-COPD in our subjects (stable, 2.59 ± 2.0 % vs. AE-COPD, 1.41 ± 0.83 %; mean ± SEM, *n* = 15 events; *p* = 0.170). By contrast, results of the clinical differential counts confirmed that the change in granulocytes we observed was due to an increase in neutrophils (stable, 70.71 ± 5.37 % vs. AE-COPD, 79.04 ± 8.29 %; p = 0.013). We found no change in numbers of platelets (stable, 266.6 ± 47.2 × 10^3^/μL vs. AE-COPD, 258.4 ± 54.4 × 10^3^/μL; *p* = 0.33).

**Fig. 3:**
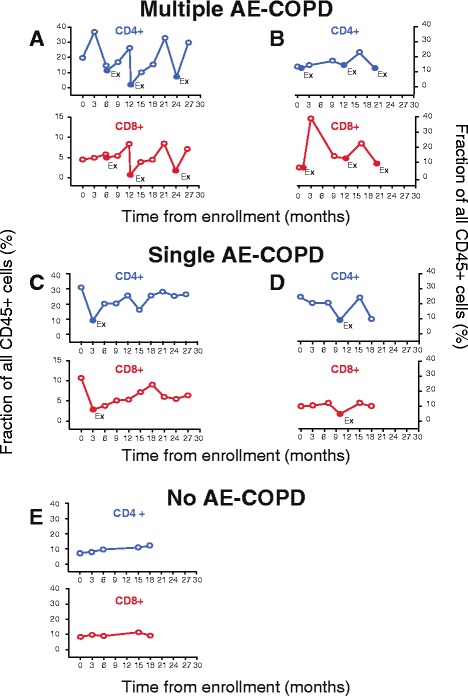
Representative graphs from individual subjects tracking percentages of CD4+ and CD8+ T cells during stable and exacerbation visits. Peripheral blood was collected during stable visits and during AE-COPD, prior to medication. Cells were stained and analyzed by flow cytometry. Panels **a**-**e** each depicted the data for a single subject; CD4+ T cells (upper panels, blue) and CD8+ T cells (lower panels, red) shown as a percentage of all CD45+ leukocytes. **a**, **b**. Two subjects with multiple AE-COPD; **c**, **d**. two subjects with a single AE-COPD; and **e**, subject with no AE-COPD. Stable visits are shown by open circles and AE-COPD are shown in solid circles labeled with “Ex”

Data from representative individuals demonstrated a recurring pattern, in which AE-COPD were marked by decreased percentages of both CD4+ and CD8+ T cells in peripheral blood, followed by a rebound in those cell types at the next stable visit (Fig. 3). Interestingly, in some subjects who had a scheduled visit shortly before a clinically-defined exacerbation (Fig. 3a, b), the decline in cell frequency could actually be observed shortly before the AE-COPD. This finding suggests that immune activation might precede symptoms, but a larger study will be needed to test that possibility. In contrast, the sole subject with no reported AE-COPD events did not display a change in T cell frequency (Fig. 3e).

### Activation markers CD40 and CD86 are increased on BDCA-1 DCs during AE-COPD

**Fig. 4:**
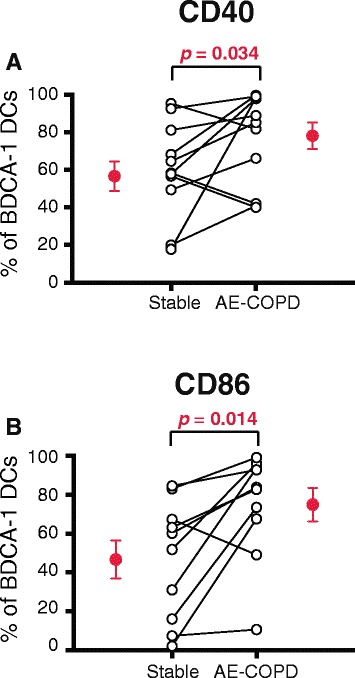
CD40 and CD86 increased on BDCA-1+ DCs from peripheral blood during AE-COPD. Peripheral blood was collected during stable visits and during AE-COPD, prior to medication (*n* = 10 paired samples). Cells were stained and analyzed by flow cytometry. After gating on BDCA-1+ DCs, the percent of cells expressing (**a**) CD40 and (**b**) CD86 was determined. Open circles represent individual subjects; mean ± SEM of the grouped data are shown by the single red circles. The Wilcoxon matched-pairs signed rank test was used to determine significant differences between visits

We also analyzed the surface expression of receptors specific to the various leukocyte populations. On both CD4+ and CD8+ T cells, we analyzed CD69, CD25, CD27, CD62L and IL-18R, which collectively contribute to defining T cell activation history. There was no difference in the percentage of T cells expressing these receptors between stable and AE-COPD visits, nor were there any significant changes in B cells or NK cells (data not shown). On the three DC population, we analyzed the activation and maturation markers CD40, CD80, CD83 and CD86. Despite an absence of change in the numbers or frequency of DCs, we found that the percentage of mDC1 that expressed CD40 and CD86 increased significantly during AE-COPD (Fig. 4). By contrast, expression of CD80 and CD83 did not change on mDC1, nor were there any significant changes in receptor expression on mDC2 or pDC (not shown).

### Increased production of specific analytes in the serum during AE-COPD

**Fig. 5:**
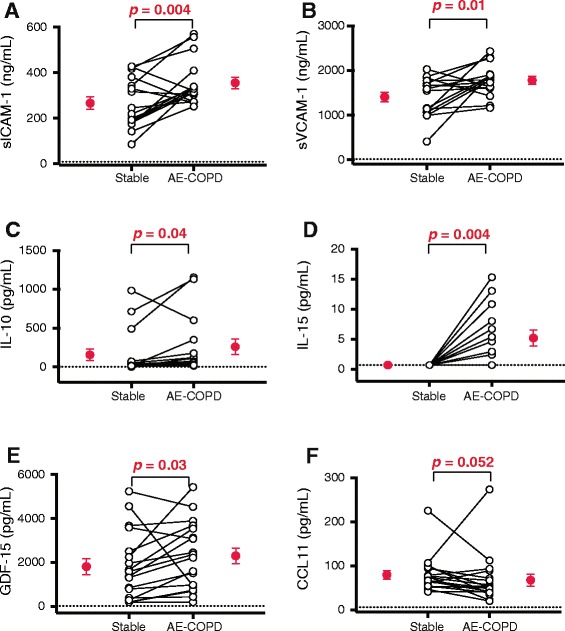
Soluble adhesion molecules and inflammatory mediators increased while CCL11 decreased in the serum during AE-COPD. Serum was collected during stable visits and during AE-COPD, prior to medication (*n* = 18 paired samples). Samples were stored at −80 °C prior to analysis by Luminex® assay, except as noted. (**a**) sICAM-1, (**b**) sVCAM-1, (**c**) IL-10, (**d**) IL-15, (**e**) GDF-15 (by ELISA), (**f**) CCL11 are shown. Open circles represent individual subjects; mean ± SEM of the grouped data are shown by the single red circles. Note differences in units of measurement. The dotted line represents the minimal detectable concentration. The Wilcoxon matched-pairs signed rank test was used to determine significant differences between visits

We analyzed serum for 41 different analytes using a combination of Luminex® and ELISA assays (Table 2). In addition to CRP, considered above, five analytes increased significantly during AE-COPD: soluble intracellular adhesion molecule (sICAM)-1, soluble vascular cell adhesion molecule (sVCAM)-1, IL-10, IL-15, and growth and differentiation factor 15 (GDF-15) (Fig. 5a-e). Serum IL-15 measurements during stable visits were at or near the limit of detection, however IL-15 levels were significantly increased in all but one of the AE-COPD events. Interestingly, CCL11 showed a trend to decrease during AE-COPD (Fig. 5f), which did not attain statistical significance (stable, 79.7 ± 41.1 pg/mL vs. AE-COPD, 67.8 ± 56.7 pg/mL; mean ± SD; *p* = 0.052).

**Table 2:** Effect of COPD exacerbation on biomarkers in human sputum and serum

		Sputum			Serum	
Biomarker	Stable (pg/mL)	Exacerbation (pg/mL)	P-value	Stable (pg/mL)	Exacerbation (pg/mL)	P-value
CCL2	462.1 ± 595.5	631.5 ± 960.4	0.52	373.6 ± 186.8	372.7 ± 227.0	1.0
CCL3	41.9 ± 27.5	58.0 ± 42.6	0.35	80.6 ± 35.8	86.7 ± 39.6	0.41
CCL4	110.8 ± 89.7	138.6 ± 116.4	0.64	128.9 ± 52.2	134.2 ± 57.3	0.64
CCL5	16.6 ± 4.0	16.9 ± 2.9	0.50	8,636 ± 2,200	8,500 ± 2,561	0.83
CCL7	16.3 ± 2.9	20.3 ± 11.5	0.44	61.1 ± 38.9	73.6 ± 56.9	0.40
CCL8	Not detected	Not detected		15.5 ± 3.8	16.5 ± 7.6	0.89
CCL11	Not detected	Not detected		79.7 ± 41.1	67.8 ± 56.7	0.06
CRP	8355 ± 12659	43812 ± 58313	0.004	0.58 ± 0.40 mg/L	6.37 ± 7.50 mg/L	0.0005
CXCL9	748.4 ± 863.8	641.5 ± 765.7	0.97	75.2 ± 32.8	84.9 ± 32.7	0.22
CXCL10	150.1 ± 101.2	568.4 ± 799.5	0.20	40.7 ± 8.9	67.8 ± 76.2	0.30
ENA-78	1544 ± 2243	573.5 ± 814.7	0.43	366.4 ± 278.8	435.6 ± 340.5	0.73
E-Selectin	Not detected	Not detected		4.5x10^4^ ± 1.9x10	4.9x10^4^ ± 1.7x10^4^	0.72
GDF-15	ND	ND		1,806 ± 1,558	2,295 ± 1,527	0.03
GRO-α	65.1 ± 72.5	52.7 ± 47.0	0.76	34.8 ± 31.1	43.7 ± 42.7	0.23
sICAM-1	1,370 ± 1,256	1,135 ± 1,118	0.81	2.6x10^5^ ± 1.1x10^5^	3.5x10^5^ ± 1.0x10^5^	0.004
IFN-α	Not detected	Not detected		Not detected	Not detected	
IFN-β	Not detected	Not detected		802.9 ± 1096	1015.7 ± 1376	0.13
IFN-γ	Not detected	Not detected		Not detected	Not detected	
IL-1β	20.3 ± 29.7	309.2 ± 567.2	0.15	Not detected	Not detected	
IL-1R1	ND	ND		Not detected	Not detected	
IL-1R2	ND	ND		Not detected	Not detected	
IL-1ra	34.9 ± 70	36.2 ± 54.7	0.43	Not detected	Not detected	
IL-6	117.1 ± 108.2	225.9 ± 193.5	0.16	11.9 ± 16.0	43.7 ± 103.6	0.21
sIL-6R	Not detected	Not detected		3.8x10^4^ ± 1.3x10^4^	4.0x10^4^ ± 2.2x10^4^	0.90
IL-8	3.7x10^6^ ± 2.6x10^6^	5.5x10^6^ ± 4.3x10^6^	0.16	33.7 ± 27.3	56.6 ± 40.0	0.16
IL-10	6.6 ± 4.7	27.6 ± 79.3	0.85	156.1 ± 299	260.0 ± 392	0.04
IL-15	Not detected	Not detected		0.7 ± 0.1	5.2 ± 5.0	0.004
IL-17	Not detected	Not detected		Not detected	Not detected	
IL-18	40.7 ± 57.6	57.6 ± 100.3	0.99	20.9 ± 9.7	27.2 ± 13.3	0.18
IL-23 p19	Not detected	Not detected		Not detected	Not detected	
MMP-9	ND	ND		8.1x10^5^ ± 4.8x10^5^	10.8x10^5^ ± 5.9x10^5^	0.37
MMP-12	9,758 ± 21,460	10,129 ± 19,529	0.85	ND	ND	
MPO	2.0x10^5^ ± 1.2x10^5^	2.5x10^5^ ± 0.6x10^5^	0.46	6.05x10^5^ ± 4.4x10^5^	5.6x10^5^ ± 4.3x10^5^	0.70
TIMP-1	381.4 ± 66.3	280.7 ± 72.9	0.016	8,973 ± 2,847	9,769 ± 2,185	0.42
TIMP-2	60.5 ± 26.1	116.9 ± 63.0	0.031	8,508 ± 13,251	8,958 ± 7,733	0.66
TIMP-3	Not detected	Not detected		1,488 ± 979	1,313 ± 794	0.83
TIMP-4	Not detected	Not detected		155.2 ± 69.3	125.1 ± 51.8	0.42
TNF-α	ND	ND		10.7 ± 5.8	16.9 ± 23.1	0.31
sTNFR1	14,681 ± 29,002	8,669 ± 15,039	0.96	1,829 ± 979	2,011 ± 570	0.27
sTNFR2	4,692 ± 6,700	9,140 ± 15,732	0.38	9,881 ± 5,189	10,488 ± 4,189	0.45
sVCAM-1	ND	ND		1.4x10^6^ ± 0.4x10^6^	1.8x10^6^ ± 0.3x10^6^	0.014
VEGF	Not detected	Not detected		Not detected	Not detected	

Data are shown as mean ± SD in pg/mL unless otherwise indicated; ND: not done

### AE-COPD leads to decreased TIMP-1 and increased TIMP-2 production in the sputum

**Fig. 6:**
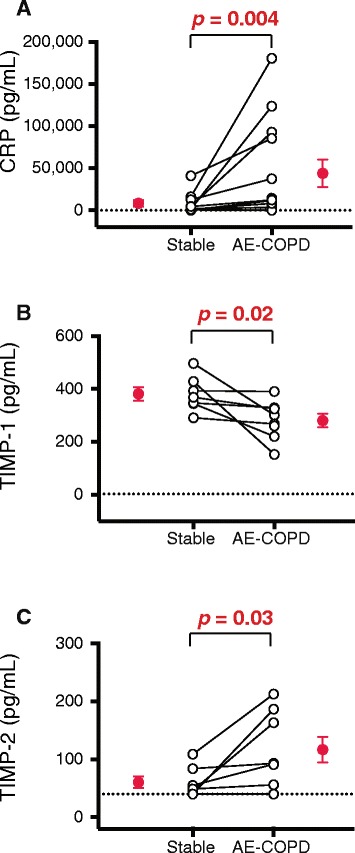
CRP and TIMP-2 increased in sputum during AE-COPD, whereas TIMP-1 decreased. Sputum was collected during stable visits and during AE-COPD, prior to medication. After processing, sputum supernatants were stored at −80 °C prior to analysis by Luminex. (**a**) CRP, (**b**) TIMP-1, (**c**) TIMP-2 are shown (*n* = 10 paired samples for CRP and *n* = 7 paired samples for TIMP-1 and TIMP-2). Open circles represent individual subjects; mean ± SEM of the grouped data are shown by the single red circles. The dotted line represents the minimal detectable concentration. The Wilcoxon matched-pairs signed rank test was used to determine significant differences between visits

We also analyzed sputum supernatants for 36 different analytes using a combination of Luminex® and ELISA assays (Table 2). Significant increases during AE-COPD were found for CRP and tissue inhibitor of metalloproteinase (TIMP)-2, while TIMP-1 levels in sputum decreased during AE-COPD (Fig. 6). IL-6 and IL-8 levels in sputum also increased slightly during AE-COPD, but did not reach statistical significance (IL-6: stable, 117.1 ± 108.2 pg/mL vs. during AE-COPD, 225.9 ± 193.5 pg/mL; IL-8: stable, 3.7×10^3^ ± 2.6×10^3^ ng/mL vs. AE-COPD, 5.5×10^3^ ± 4.3×10^3^ ng/mL; mean ± SD, *p* = 0.16 for both analytes).

## Discussion

This observational study of paired samples from COPD subjects in the stable and exacerbated states demonstrates that CD4+ T cells and CD8+ T cells decreased in peripheral blood during AE-COPD. Decreases occurred both in absolute numbers and as a percentage of all leukocytes, and were noted repeatedly in those with multiple AE-COPD. We found no changes during AE-COPD in numbers or percentages of B cells, NK cells or DCs in peripheral blood, although numbers of neutrophils increased, as did expression of CD40 and CD86 by BDCA-1+ DC, compatible with activation. We also demonstrate significant increases during AE-COPD in serum levels of GDF-15, a sensitive marker of cardiopulmonary stress that in other settings predicts reduced long-term survival, and of IL-10, IL-15, sICAM and sVCAM. These results extend the concept that AE-COPD are systemic inflammatory events to which adaptive immune mechanisms contribute.

Determining whether lymphocyte recruitment should be targeted to improve AE-COPD clinical outcomes requires defining whether lung inflammation depends on that recruitment, or conversely can be mediated by the newly recognized and sizeable populations of lung resident lymphocytes [23, 24]. As assessed by bronchoalveolar lavage, CD4+ T cells are reduced in the lungs of active smokers without airflow obstruction [25] and in active smokers regardless of airflow obstruction [26], whereas bronchial biopsies showed increased intraepithelial CD4+ T cells in COPD patients, relative to smokers and never-smokers [27]. In terms of function, we recently showed that lung resident CD4+ T cells in many subjects with stable advanced COPD have very reduced ability to produce inflammatory cytokines, either spontaneously or on polyclonal stimulation, relative to subjects without COPD [28]. Thus, the CD4-dependent component of an AE-COPD should depend heavily on acute recruitment, and on subsequent activation by lung resident DC. Such activation is likely to be intact, because during clinical stability expression of co-stimulatory surface receptors by all three lung DC populations increases in correlation with spirometrically-defined COPD progression [29, 30]. Moreover, lung DC from COPD can polarize peripheral blood CD4+ T cells in vitro [31], suggesting a similar role in the lungs.

By contrast, relative to lung CD8+ T cells from smokers with preserved spirometry, lung resident CD8+ T cells from COPD subjects have greater capacity to secrete proinflammatory cytokines such as IFN-γ and TNF-α [11, 12]. Because such secretion can be triggered by IL-18 without the need for interaction with lung DC [11], lung resident CD8+ T may not only respond appropriately to cognate antigens (e.g., virally-infected lung epithelium), but might also indiscriminately sustain inflammation through bystander activation. Hence, the lung resident CD8+ T cell population, which increases in extent with worsening FEV1 [13, 32, 33], appears quite capable of stimulating lung damage by activating macrophages and by recruiting other inflammatory cell types. However, the peripheral blood of COPD patients also contains an increased proportion of IFN-γ + and TNF-α + CD8+ T cells, relative to healthy never-smokers [34]. Therefore, establishing whether the contribution of CD8+ T cells to inflammation in AE-COPD also depends in part on cells acutely recruited from the blood will be an important future goal.

The large body of experimental data on lymphocyte trafficking [35] implies that the decreases we found in blood CD4+ and CD8+ T cells during AE-COPD reflect recruitment both of naïve and memory lymphocytes into regional lymph nodes and organized lymphoid tissues (which develop within lung parenchyma in advanced COPD [33, 36]) and of effector-memory cells to the lungs. The finding that blood T cell expression of activation markers showed no net change might reflect these competing patterns of migration. It contrasts with evidence of T cell activation on presentation during severe asthma attacks [37]; the same study found that blood CD4+ T cells of five subjects hospitalized for AE-COPD showed increased expression of HLA-DR, which we did not measure, but not of CD25, in agreement with our data [37]. The disparity with the acute asthmatics might relate to the lesser degree of acute stress in our subjects, despite the severity of their underlying lung function in the stable state, or may be due to differences between the two diseases.

Our results in peripheral blood complement two studies showing increased percentages of total lymphocytes (defined by cytospin differential counts) in sputum in this setting [14, 15]. Collectively, these two studies and ours imply that T cell recruitment to the lungs is a component of AE-COPD, without proving causality in pathogenesis. By contrast, no change in percentages of lymphocytes in sputum or in density of CD4+ or CD8+ T cells in endobronchial biopsies was seen in an earlier study, which did, however, find a significant increase during exacerbation in airway wall numbers of CD3+ T cells [16]. Another study of sputum during AE-COPD found decreased CD4:CD8 ratios (assessed by immunofluorescent staining of cytospins), relative to paired samples in the stable state, but no change in percentages of lymphocytes (assessed morphologically) among total sputum leukocytes [17]. Thus, controversy remains about T cell recruitment to the lungs during AE-COPD.

We cannot address this controversy with our own sputum data. At the time of this study, we were not yet using flow cytometry to analyze sputum leukocyte subsets, as we are now doing successfully in stable COPD [20]. Our preliminary results suggest that to quantify lymphocytes in respiratory secretions accurately, flow cytometry will be superior to analysis of cytospins. This question is being examined prospectively in the Subpopulations and Intermediate Outcome Measures in COPD study (SPIROMICS) [38]. Supporting this possibility, paired comparisons of differential counts in bronchoalveolar lavage samples from humans and mice showed that cytospins result in preferential loss of lymphocytes, relative to more gentle sample preparation [39, 40]. Because flow cytometry also extends the identification of lymphocyte subsets and their activation states in sputum or other samples well beyond the capability of Giemsa-stained cytospins, we believe that it has considerable promise to investigate multiple airways diseases.

Our demonstration that serum GDF-15 levels rose in uncomplicated AE-COPD treated successfully in outpatients is significant, as this molecule has emerged as a key biomarker of cardiopulmonary stress, especially involving pulmonary vasculature. GDF-15 is a divergent TGF-beta superfamily member, first cloned from activated macrophages [41] and named Macrophage inhibitory cytokine-1 (MIC-1). However, the same gene was cloned by other strategies, leading to many alternative names [42–45]. GDF-15 plays key regulatory roles in processes as diverse as cell cycle progression, differentiation, maintenance of pregnancy, apoptosis and tumor progression [46, 47]. Serum levels of GDF-15 levels rise modestly in acute coronary syndrome and congestive heart failure, in both conditions accurately predicting one-year mortality [48–50], but are very elevated in acute pulmonary embolus [51] and pulmonary hypertension [52–54]. Elevated GDF-15 levels also predicted long-term mortality in a population-based Swedish male cohort (adjusted odd-ratio of death of 3.38, 95 % CI 1.38–8.26), independently of baseline IL-6 or CRP levels [55]. Hence, this molecule merits additional study as a COPD biomarker.

The elevation we demonstrated in serum IL-15 is significant due to its multiple actions that could potentiate lung inflammation during AE-COPD (reviewed in [56]). These include antigen-independent activation of CD8+ T cells and enhanced effector function of CD8+ T cells, NK cell and NK T cells. IL-15 also attracts and activates neutrophils, while promoting their retention and delaying apoptosis. This last action is shared with multiple types of leukocytes and respiratory epithelial cells, and might explain the previous observations that the fraction of circulating apoptotic neutrophils is deceased during AE-COPD [57, 58], although that finding might also reflect altered clearance. The lack of a parallel change in IL-15 expression in sputum might indicate that its source is not within the lungs, that its elaboration during AE-COPD is directed away from the airway lumen, or potentially that some factors during inflammation inhibited its immunodetection. Dissociation of biomarker concentrations between the peripheral blood and sputum compartments, reflected in our measurements of other biomarkers, is not unprecedented. A study analyzing the expression during the stable state of 100 biomarkers (including IL-15) in bronchoalveolar lavage, bronchial biopsies, serum and induced sputum from healthy smokers (n = 23) and COPD subjects (n = 24) observed no correlations between lung and serum biomarkers [59]. Additional investigation in larger cohorts is needed to determine the most suitable biomarkers to define the pathologic inflammatory processes occurring within the airways during AE-COPD, which may vary with the cause of specific episodes.

Multiple other circulating biomarkers of inflammation are upregulated during AE-COPD (reviewed in [60]). Although our finding of increased sVCAM is novel to our knowledge, the elevations detected in CRP, IL-10 and sICAM agree with some [22, 61, 62] but not all studies [63]. We also observed an increase in mean serum levels of both IL-6 and IL-8 during AE-COPD, as has been seen by some [22, 62, 64], but not all studies [9]; however, neither attained statistical difference from stable visits, likely due to our sample size. We did not see increased MPO, TNF-α, or TNF receptors I and II during AE-COPD, which others have reported.

Induced or spontaneously produced sputum has also been analyzed extensively during AE-COPD, with elevations previously shown for IL-1β, IL-6, TNF-α, TNF receptors I and II, CCL5, CCL4 [9], LTB4 [65] and MMP-9 [14]. We found marked but non-statistically significant elevations in sputum IL-1β concentrations and a non-significant trend towards higher CXCL10 in serum, which a previous analysis determined to be the two biomarkers that individually best discriminated bacterial AE-COPD from viral AE-COPD, respectively [9]. The broad range of these measures in our study could be taken to imply heterogeneous etiologies; because we did not perform microbiological analysis in this study, we cannot address that possibility. The current finding of reduced sputum TIMP-1 during AE-COPD, the only sputum analyte to decrease, agrees with a previous study [14]. By contrast, we noted increased TIMP-2, another inhibitor of MMPs. It is uncertain why the two TIMPs differed, especially as they are produced by multiple parenchymal lung cell types, including bronchial epithelial cells, type II alveolar pneumocytes and smooth muscle cells, plus by neutrophils and macrophages [66]. The functions of TIMP-1 and TIMP-2 during AE-COPD are unknown. Although neither has been directly implicated in lymphocyte recruitment to the lungs, TIMP-1 does appear to regulate CD4+ T cell migration into brain parenchyma in a murine model of viral encephalitis, independently of MMPs [67]. Furthermore, new data is constantly emerging on the ability of TIMPs to affect many other cellular functions, such as growth, migration, and apoptosis [68]. We did not see increases in any sputum biomarkers known to drive lymphocyte recruitment, which may be due to a lack of power, but might also argue that lymphocytes are not recruited to the lungs during AE-COPD. However, two inter-related alternatives should be considered: first, that chemokines responsible for lymphocyte recruitment into lung parenchyma might not be elevated in sputum due to preferential abluminal secretion and binding to matrix; second, that lymphocytes are recruited into lung parenchyma and especially intra-epithelial sites during AE-COPD, but do not transmigrate into airways and so cannot be detected in sputum. The latter possibility merits testing.

Strengths of the current study include the rigorous definition of AE-COPD as an event requiring a clinical decision to treat with steroids, antibiotics or both by a physician blinded to the biomarker data. The chief limitation is the small number of subjects having paired samples from stable and exacerbation visits. Based on the highly productive experience of the East London cohort, we had originally planned a 100 subject cohort. However, shortly after initiation of this study, both our sites joined the NHLBI-sponsored trial of macrolide therapy to prevent AE-COPD [69], which had similar entry criteria. Many potential subjects preferred to enroll in that interventional trial, and we had limited success in crossing over subjects on completion of their year of study drug. Additionally, 13 of our subjects had at least one self-reported AE-COPD for which they sought medical attention locally rather than coming for a study visit.

## Conclusions

During event-defined AE-COPD, relative to paired samples collected during clinical stability, neutrophils increased while CD4+ and CD8+ T cells (but not B cells, NK cells or DC) decreased in peripheral blood, both relatively and absolutely and in significant correlation with serum CRP levels. There were also significant increases in serum concentrations of GDF-15, a biomarker of pulmonary vascular stress with known strong independent prognostic value for long-term survival, and of sICAM-1, sVCAM-1, IL-10 and IL-15. These findings support the growing evidence that dysregulated inflammation contributes to the morbidity of AE-COPD and their sustained adverse effect on survival.

During review of this article, GDF-15 was reported to be elevated in 29 subjects hospitalized for AE-COPD, relative to a separate group of subjects with stable COPD and to healthy control subjects [70].

## Additional file

Additional file 1:
**Supplemental materials & methods.**

## Abbreviations

AE-COPD: Acute exacerbation of chronic obstructive pulmonary disease
BCSS: Breathlessness, cough, and sputum scale
COPD: Chronic obstructive pulmonary disease
CRP: C-reactive protein
DC: Dendritic cells
FEV_1_: Forced expiratory volume in 1 s
FVC: Forced vital capacity
GDF-15: Growth and differentiation factor 15
ICS: Inhaled corticosteroids
NK: Natural killer
sICAM-1: soluble intracellular adhesion molecule-1
sVCAM-1: soluble vascular adhesion molecule-1
TIMP: Tissue inhibitor of metalloproteinase
UMHS: University of Michigan Health System
VAAAHS: Veterans Affairs Ann Arbor Healthcare System
