# Supplementary material for: Acute exacerbations of chronic obstructive pulmonary disease are associated with decreased CD4+ & CD8+ T cells and increased growth & differentiation factor-15 (GDF-15) in peripheral blood
Source: Respir Res. 2015 Aug 5;16(1):94. doi: 10.1186/s12931-015-0251-1 (PMC4531816; doi:10.1186/s12931-015-0251-1)
Supplement: Additional file 1: — Supplemental materials & methods. [file 12931_2015_251_MOESM1_ESM.docx]

# Supplemental materials & methods

## Flow cytometry

We used monoclonal antibodies against the following surface antigens (clones shown in parentheses): CD3 (HIT3a), CD4 (OKT4), CD8 (HIT8a), CD15 (HI98), CD19 (H1B19), CD25 (BC96), CD27 (O323), CD40 (LOB7/6), CD45 (HI30), CD56 (N-CAM), CD69 (FN50), CD80 (2D10.4), CD83 (HB15e), CD86 (IT2.2), CD123 (6H6), HLA-DR (LN3) and IL-18R (H44) (eBioscience, San Diego, CA); CD62L (Dreg 56) (BD Biosciences, San Jose, CA); CD1c/BDCA-1 (AD5-8E7), CD141/BDCA-3 (AD5-14H12) and CD303/BDCA-2 (AC144) ( (Miltenyi Biotec, San Diego, CA). Antibodies and isotype-matched controls were directly conjugated to either fluorescein isothiocyanate (FITC), phycoerythrin (PE), phycoerythrin- cyanine 7 (PE-Cy7), allophycocyanin (APC), Pacific Blue, Alexa Fluor 700, or biotin, with the biotinylated antibodies developed using streptavidin-phycoerythrin-cyanine 5 (SA-PE-Cy5).

Whole blood was analyzed by flow cytometry for the following cell populations: dendritic cells (DCs), granulocytes, B cells, CD4+ T cells, CD8+ T cells and natural killer (NK) cells. To identify DCs, we first gated on non-autofluorescent CD45+ leukocytes that were CD3- and CD19-. Myeloid DCs (mDC) were then identified as either HLA-DR+, CD1c/BDCA-1+ cells (mDC1) or HLA-DR+, CD141/BDCA-3+ cells (mDC2), while plasmacytoid DCs (pDC) were identified as CD123+, CD303/BDCA-2+ cells [[29](#_ENREF_29), [70](#_ENREF_70)]. Granulocytes were selected using CD45+ and CD15+ positive staining plus high side scatter. For B cells, CD4+ T cells, CD8+ T cells and NK cells, we first gated on CD45+ leukocytes, than set a lymphocyte gate using forward scatter and side scatter, and finally identified cells as either CD19+, CD3+ CD4+, CD3+ CD8+, or CD3- CD56+, respectively.

## Serum and sputum protein measurements

Serum and sputum samples were stored at -80°C until all could be analyzed simultaneously. To analyze protein concentrations, we used two methodologies. First, we used a Luminex 200 system (Luminex Corporation, Austin, TX), according to manufacturers’ instructions and multiplex bead sets from the indicated vendors to measure the following analytes: CCL2 (MCP-1), CCL3 (MIP-1α), CCL4 (MIP-1β), CCL5 (RANTES), CCL7 (MCP-3), CCL8 (MCP-2), CCL11 (Eotaxin), C-reactive protein (CRP) (used for sputum specimens only), CXCL1 (GRO-α), CXCL9 (MIG), IFN-α, IFN-γ, IL-6R (Life Technologies, Grand Island, NY); CXCL10 (IP-10), E-selectin, IL-1β, IL-1R1, IL-1R2, IL-1ra, IL-6, IL-10, IL-15, IL-17, matrix metalloproteinase (MMP)-9, MMP-12, MPO, soluble (s) ICAM-1, sVCAM-1, TNF-α, TNFR1, TNFR2, VEGF (EMD Millipore); CXCL5 (ENA-78), IL-8, tissue inhibitor of metalloproteinases (TIMP)-1, TIMP-2, TIMP-3 and TIMP-4 (R&D Systems, Minneapolis, MN).

We employed ELISAs used according to manufacturers’ instructions to measure protein concentration of the following analytes: GDF-15 (R&D Systems), IL-18 (Life Technologies), IL-23p19 (eBioscience, San Diego, CA) and IFN-β (PBL Biomedical, Piscataway, NJ).
